# Extraction and Characterization of Tamarind (*Tamarind indica* L.) Seed Polysaccharides (TSP) from Three Difference Sources

**DOI:** 10.3390/molecules21060775

**Published:** 2016-06-15

**Authors:** Khanittha Chawananorasest, Patsuda Saengtongdee, Praphakorn Kaemchantuek

**Affiliations:** Thailand Institute of Scientific and Technological Research (TISTR), 35 Mu 3 Tambon Khlong Ha, Amphoe Khlong Luang, Pathum Thani 12120, Thailand; new_ninsho@hotmail.com (P.S.); praphakorn@tistr.or.th (P.K.)

**Keywords:** tamarind seed polysaccharide (TSP), *Tamarind indica* L., natural polysaccharides

## Abstract

Tamarind seed polysaccharide (TSP), a natural polysaccharide extracted from tamarind seeds is used in the pharmaceutical, textile and food industries as a mucoadhesive polymer. This work aimed to extract TSP from tamarind seeds from three sources with two methods and characterized its physical and chemical properties. Kernel powder of tamarind seeds was slurried into a clear solution, set aside overnight and then centrifuged at 6000 rpm for 20 min to separate all foreign matter. The supernatant was separated and poured into excess 95% ethanol with continuous stirring. The precipitate obtained was collected and dried in the oven and then the dried TSP polymer was stored in a desiccator. The dried TSP was analyzed by ^1^H-NMR, FT-IR and XRD. The results showed TSP from tamarind seeds taken from paddy farmland (A), a waste from the export tamarind juice industry (B) and the export tamarind powder industry(C) gave yields of 31.55%, 26.95% and 17.30%, respectively, using method 1 and 11.15%, 53.65% and 54.65%, with method 2, respectively, but method 2 gave purer TSP than method 1. The FT-IR spectra displayed peaks at 3351.95 cm^−1^, 2920.76 cm^−1^, 1018.85 cm^−1^ and 555.16 cm^−1^. The ^1^H-NMR showed polysaccharide peaks between δ 3.50–4.20 ppm and XRD diagrams indicated their amorphous nature. Future works will focus on the quantitative analysis, biological activity and possible use of TSP as a drug delivery system.

## 1. Introduction

**Figure 1:**
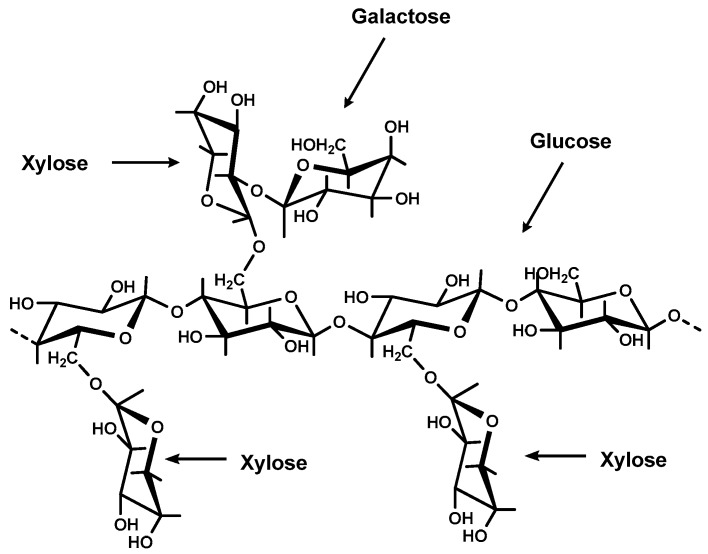
Structure of tamarind seed polysaccharide (TSP).

Natural polymers or gums have been used in the preparation of release and controlled release drug dosage forms, because of their great properties, such as biodegradability, non-toxicity, biocompatibility in Nature and swelling when they come in contact with aqueous media. Tamarind (*Tamarind indica* L.) belongs to the Leguminosae family [1]. The oil extracted from its seeds is rich in eicosanoic fatty acids such as palmitic, oleic and linoleic, the highest concentrations corresponding to linoleic acid and palmitic acid, present in 36%–49% and 14%–20%, respectively [2]. Tamarind seed polysaccharide (TSP), is a natural branched polysaccharide polymer with a molecular weight of 700–880 kDa [3]. TSP constitutes about 65% of the tamarind seed composition [4]. TSP is composed of a (1→4) β-d-glucan backbone substituted with side chains of α-d-xylopyranose and β-d-galactopyranosyl linked (1→2)-α-d-xylo-pyranose linked (1→6) to glucose residues [5] (Figure 1). The chemical constituents of TSP are glucose, xylose and galactose in a ratio of 2.80:2.25:1.00 [6]. TSP, regarded as a galactoxylloglucan, is a novel polymer with various properties useful to the textile, food, and pharmaceutical industry. Singh *et al.* found that tamarind gum was a highly viscous, mucoadhesive and biocompatible natural polymer, which could be used for oral controlled drug release, ocular drug delivery systems and in the design of sustained release drug delivery systems and dosage forms [7]. TSP possesses various features, making it an attractive candidate as a vehicle for ophthalmic medicaments [8]. Mixtures of TSP and hyaluronic acid are employed as artificial tears for ophthalmic application in dry eye syndrome [9]. TSP can be used in drug delivery systems for the ocular administration of hydrophilic and hydrophobic antibiotics [10]. Tamarind seed polysaccharide is composed of pectin with a high methoxyl content (6.8%–8.37%), that promotes gel strength and heat stability [11]. It possesses properties of high viscosity, broad pH tolerance, noncarcinogenicity, mucoadhesive nature and biocompatibility [12]. And is insoluble in organic solvents and dispersible in warm water to form a highly viscous gel as a mucilaginous solution [13,14]. TSP possesses the characteristic property of forming gels with sugar concentrates in a wide pH range that are also not affected by boiling in a neutral aqueous solution, even if boiled for a long period, which makes them superior to fruit pectins [15]. It has been described as a viscosity enhancer showing mucomimetic and mucoadhesive ability to form hydrogels. The individual components of the seeds have not been fully identified and quantitated [16]. Therefore, this project aimed to extract TSP from tamarind seeds from three different sources and characterize its physical and chemical properties for the possible future development of drug delivery systems. 

## 2. Results and Discussion

The tamarind seeds were processed by separating the brown peels from the kernel seeds with a blender and separating the seeds using a plastic sieve. The kernel powder (20 g) from seeds taken from paddy farmland (A), a waste from the export tamarind juice industry (B) and the export tamarind powder industry (C) were extracted and precipitated using the two methods provided to give the dried TSP as described in Table 1.

**Figure 2:**
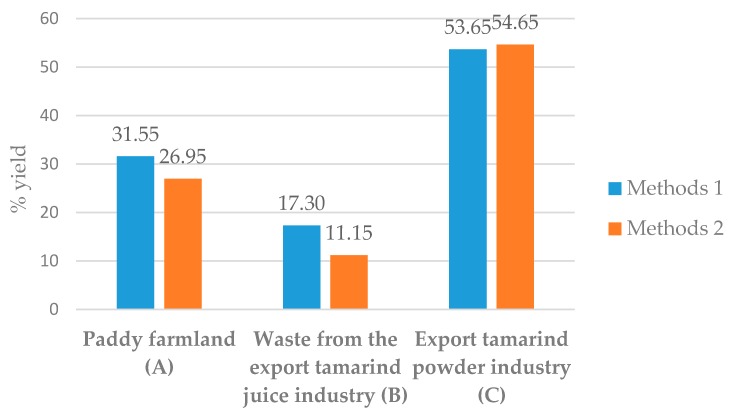
Percent yield of TSP compared to the different three sources with the two methods used.

With method 1, TSP (C) gave the highest % yield which was nearly double the amount of TSP (A) while TSP (B) gave the lowest percentage compared to the others. Similar results were seen with method 2. Comparison between the two methods indicated that method 1 gave higher TSP yields than method 2, although TSP (C) yielded about the same quantity with both methods, as shown in Figure 2. 

**Figure 3:**
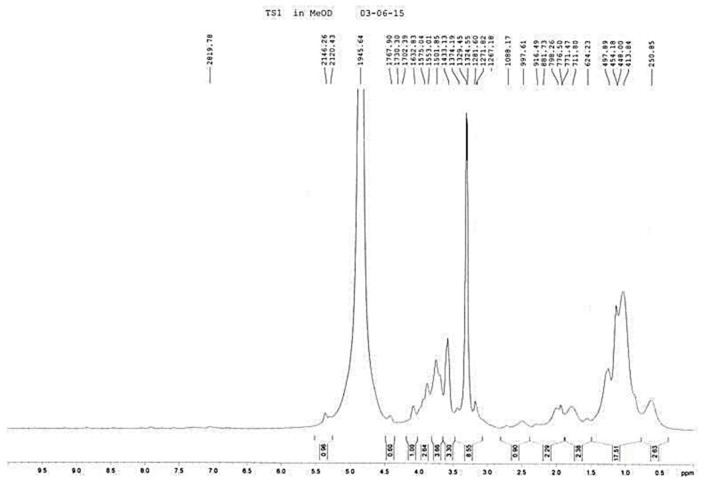

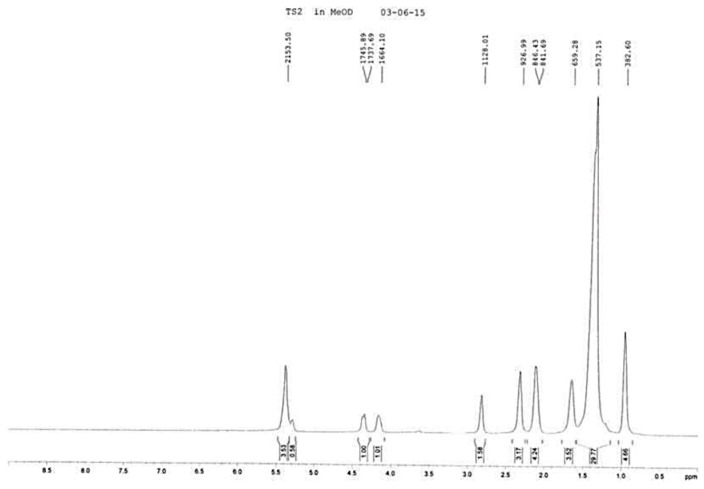
^1^H-NMR spectra in MeOD of TS1 (**top**) and TS2 (**bottom**) from Sample (A).

This can be explained by the fact that TSP (C) contained less fatty acid than that from sources A and B, respectively. The TSP from method 2 was considered to be purer than that extracted by method 1. The tamarind seeds taken from paddy farmland (A) were submitted to extraction with methanol by Accelerated Solvent Extraction (ASE), to give the methanol extract TS1. Its chemical constituents were identified by ^1^H-NMR technique, which showed TSP peaks in the δ 3.00–4.20 ppm chemical shift region and methyl and methylene groups at δ 0.45–2.00 ppm (Figure 3). Meanwhile, the ethanol extract (TS2) showed a mixture of methyl linoleate and triacylglycerol (Figure 3). The tamarind seeds powder should be extracted with methanol solvent in order to give TSP, indicated by the resonances at δ 3.00–4.20 ppm, but it must be further purified to remove the compounds responsible for the alkyl groups at δ 0.45–2.00 ppm to yield a pure TSP. On the other hand, when extracted with ethanol, only fatty acid derivatives were obtained and no TSP was present.

**Figure 4:**
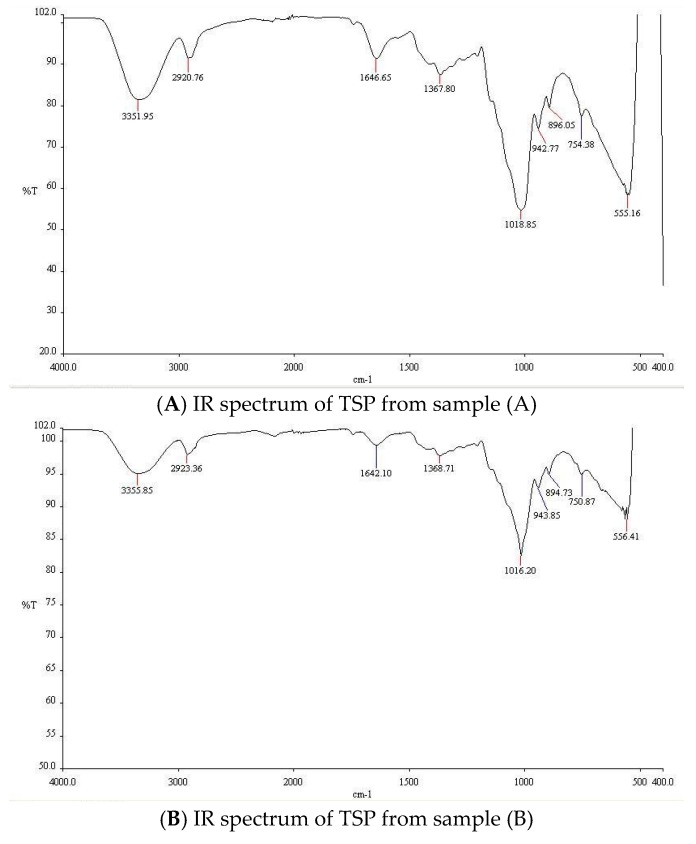

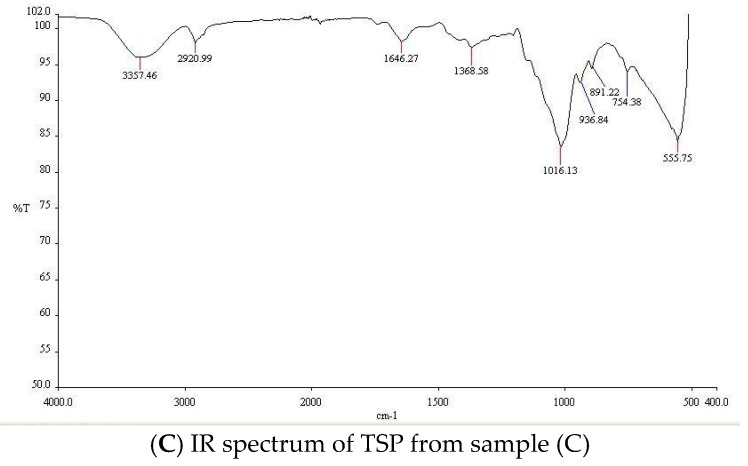
IR spectra of TSP from samples (A)–(C).

The infrared (IR) spectra of TSP extracted from the tamarind seeds taken from paddy farmland (A) (Figure 4A), a waste from the export tamarind juice industry (B) (Figure 4B), and the export tamarind powder industry (C) (Figure 4C) were recorded. TSP displayed characteristic broad peaks at 3351.95 cm^−1^, 3355.85 cm^−1^ and 3357.46 cm^−1^, respectively, representing the hydroxyl (OH) stretching of the glucan backbone.

Peaks at 2920.76 cm^−1^, 2923.36 cm^−1^ and 2920.99 cm^−1^, respectively, were attributed to alkane C–H stretching. Peaks appearing at 1018.85 cm^−1^, 1016.20 cm^−1^ and 1016.13 cm^−1^, respectively, represented (C–O–C) stretching of cyclic ethers. Peaks at 555.16 cm^−1^, 556.41 cm^−1^ and 555.75 cm^−1^, respectively, confirmed the OH bending.

**Figure 5:**
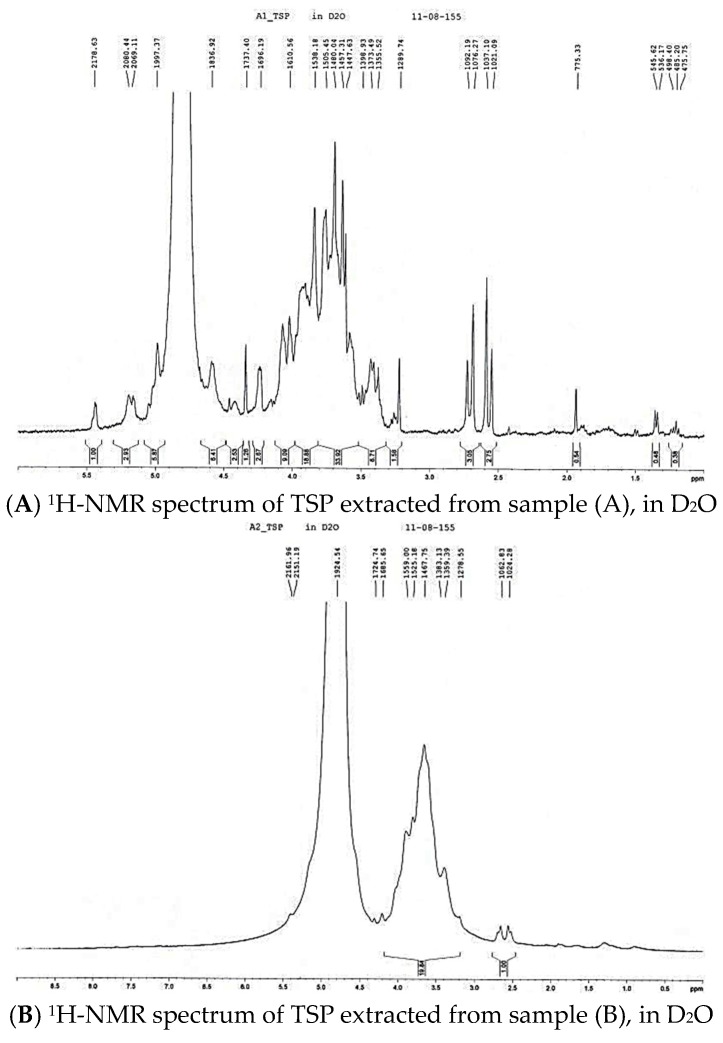

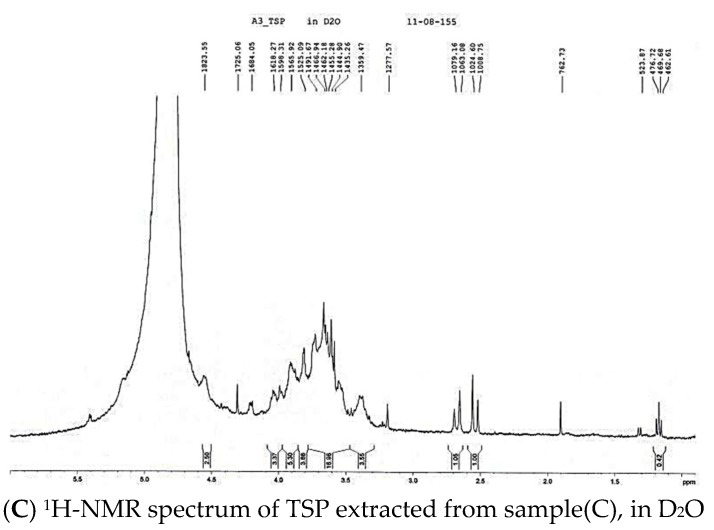
NMR spectra of TSP from samples (A)–(C).

The ^1^H-NMR spectra of the TSP extracted from tamarind seeds taken from paddy farmland (A) (Figure 5A), exported tamarind juice industry (B) (Figure 5B), and tamarind powder from the exported tamarind powder industry (C) (Figure 5C) exhibited the characteristic peaks of polysaccharides between δ 3.4–4.1 ppm. TSP (C), similarly to TSP (A), presented these chemical shifts; while the TSP (B) spectrum showed no α-residue doublet signal at δ 5.1–5.25 ppm and an absence of the singlet signal at δ 1.96 ppm, which was related to the methyl groups of rhamnose.

The ^1^H-NMR spectra of mucilage indicated that certain sugar peaks around δ 3.65–3.55 ppm can be attributed to the OH and CH group of mannose. The signals between δ 3.90–3.50 ppm correspond to the CH2 groups of arabinose. The singlet at δ 1.96 ppm is related to methyl groups and the proton linked to C-6 (δ 3.65 and δ 3.70 ppm) of rhamnose and C-4 (δ 3.98–4.28 ppm) of galactose. The anomeric protons have been assigned to the α-residue doublet signal at δ 5.1–5.25 ppm. The mucilage signals resonating at δ 4.02 and δ 3.84 ppm were assigned to H-1 of glucose. The ^1^H-NMR spectra showed a crowded signal region between δ 3.00 ppm to δ 5.00 ppm typical of polysaccharides, which confirms the presence of many similar sugar residues. The signals present between δ 3.20–4.30 ppm can be assigned to non-anomeric protons (H2-H6) whereas signals between δ 4.30 ppm to δ 4.80 ppm and δ 4.90 to δ 5.50 ppm can be suggested to correspond to the α-anomeric and β-anomeric protons, respectively. Overall, the NMR data suggest the higher purity of some samples, indicating they are perhaps more suitable for use in drug delivery systems.

**Figure 6:**
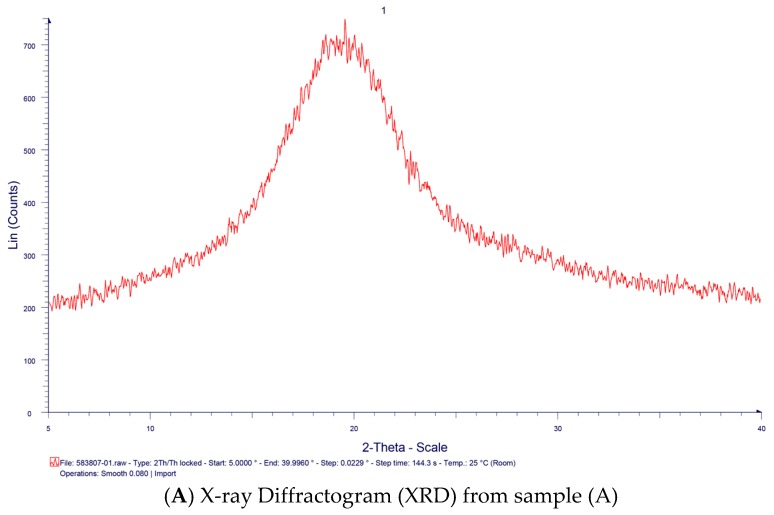

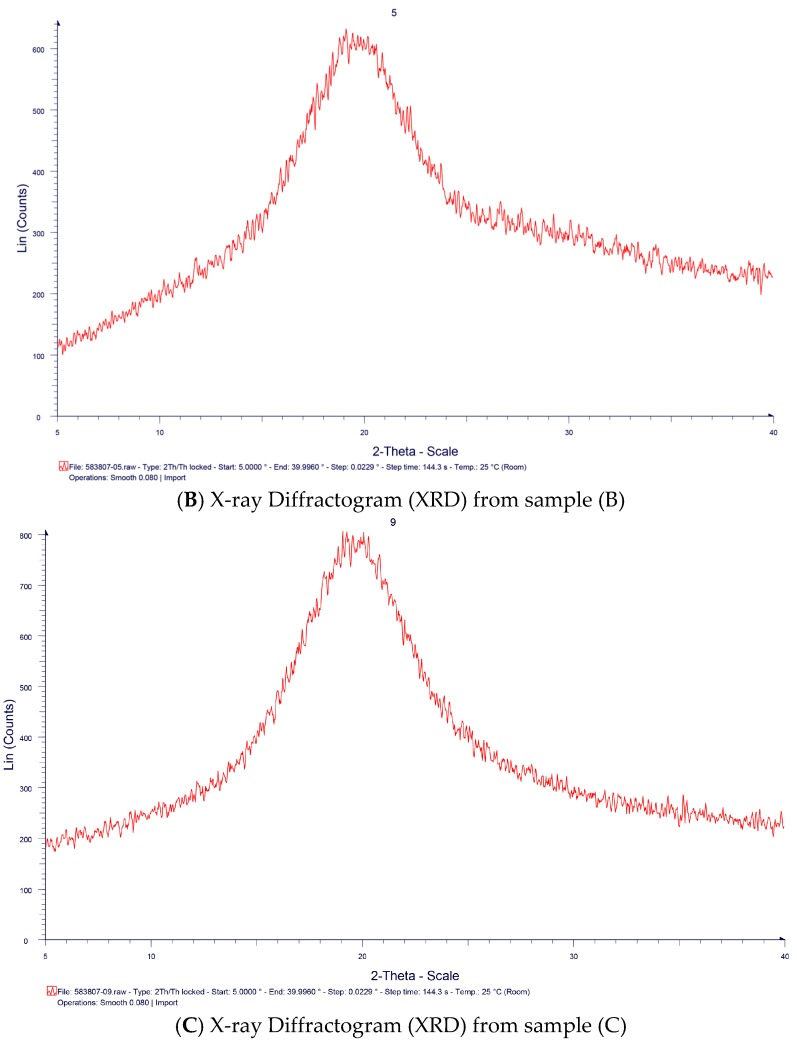
X-ray Diffractogram (XRD) spectra of TSP samples (A)–(C).

Figure 6A–C present the X-ray diffractograms of TSP extracted from a paddy farm (A), waste from the export tamarind juice industry (B), and export tamarind powder industry (C), which are typical of amorphous materials with no sharp peaks.

## 3. Materials and Methods

### 3.1. Materials

Tamarind seeds taken from paddy farmland (A) were bought from Winai Jattanakul, Phetchaboon Province, Thailand. TSP waste from the export tamarind juice industry (B) was bought from P. Prateeptong Best Foods Company Limited, Samutsongkram Province, Thailand and the TSP from the export tamarind powder industry (C) was a gift from G.M. Ichihara (Pathumthani, Thailand) Company Limited, Pathumthani Province, Thailand. The extractions used AR grade methanol and ethanol from RCI Labscan Asia, Samutprakan, Thailand.

### 3.2. TSP Extraction Procedure

#### 3.2.1. Tamarind Seed Preparation

Tamarind seeds taken from paddy farmland (A), with pulse tissue, were separated from their pulse by hand, then the seeds were washed with tap water and dried in an oven at 100 °C for 30 min. The seeds were allowed to cool down to room temperature and then lightly ground for 0.5–1 min in a blender to separate the brown peels from the kernel seeds. The kernel seeds were finally ground into powder with a blender.

#### 3.2.2. TSP Extraction

Method 1: Cold distilled water (200 mL) was added to TSP powder (20 g) to prepare a slurry. The slurry obtained was poured into boiling distilled water (800 mL) and then boiled for 20 min on a hot plate to give a clear solution that was stored overnight. The thin clear solution was further centrifuged at 6000 rpm for 20 min to separate all the foreign matter. The supernatant was separated and poured into excess 95% ethanol with continuous stirring. The obtained precipitate was collected using a stainless sieve, and dried in an oven at a temperature 50 °C for 4 h. The dried polymer was stored in a desiccator. In the same way, tamarind seed powder, waste from the export tamarind juice industry (B) and the export tamarind powder industry (C) were extracted using the procedure mentioned above. Only tamarind seeds taken from paddy farmland (A), were extracted by Accelerated Solvent Extraction (ASE) using methanol as a solvent, following by ethanol, at a temperature of 100 °C for 30 min to give methanol extract (7.51%) and ethanol extract (3.31%).

Method 2: Tamarind seed powder (50 g) was defatted using hexane and 20 g of the defatted seed powder were taken as the starting material and subjected to the process described as Method 1.

### 3.3. Methods

Tamarind seeds taken from paddy farmland (A) were extracted using a model ASE350 Accelerated Solvent Extraction (ASE) instrument (DIONEX Softron GmbH, Germering, Germany). The TSP extracts were dried in a Termaks oven (Shine Engineer International Company Limited, Bangkok, Thailand). 

### 3.4 Characterization of TSP

The structure of the TSP was analyzed by NMR using an AVANCE 400 instrument in D_2_O (Bruker, Karlsruhe, Germany). TSP samples from the three sources were subjected to FT-IR spectroscopy as KBr pellets in a range of 4500–500 cm^−1^ on a Fourier-transform infrared spectrophotometer (Spectrum 100, Perkin Elmer, Waltham, MA, USA). 

## 4. Conclusions

TSP extracted from the export tamarind powder industry (C) gave a higher yield than the TSP from the tamarind seeds taken from paddy farmland (A) or from the export tamarind juice industry (B), respectively. TSP extracted by Method 2 resulted in less fatty acid than in those prepared using Method 1. The physical and chemical property characterization of the TSP samples indicated that tamarind seed powder taken from paddy farmland (A) contained a mixture of methyl linoleate and triacylglycerol in the ethanol extract obtained by ASE, while its methanol extract yielded pure TSP, as indicated by the peaks resonating at δ 3.00–4.20 ppm in the corresponding NMR spectrum. Therefore, tamarind seed powder should be extracted with methanol rather than ethanol to obtain pure TSP. The IR showed the characteristic stretching and bending bands of the glucan backbone of TSP. The FT-IR and ^1^H-NMR confirmed the presence of a glucan backbone, non-reducing sugars and demonstrated the presence of mucilage. The TSP from (A) could be more suitable for a drug delivery system than TSP from (C) and (B) as it gave the highest TSP signals. Further work will focus on TSP sample mucohesive strength, its biological activities, toxicity evaluation and the design of controlled release drug delivery systems.

## Figures and Tables

**Table 1:** Tamarind Seed Polysaccharides (TSP) extracted from the three sources by two methods.

Methods		Tamarind Seed Sources		
		Paddy Farmland (A)	Waste from the Export Tamarind Juice Industry (B)	Export Tamarind Powder Industry (C)
Methods 1	Weight (grams)	20.00	20.00	20.00
	Supernatant (mL)	173.75	202.50	208.00
	TSP Weight (grams)	6.31	3.46	10.73
	% yield	31.55	17.30	53.65
Methods 2	Weight (grams)	20.00	20.00	20.00
	Supernatant (mL)	222.75	239.50	173.25
	TSP Weight (grams)	5.39	2.23	10.93
	% yield	26.95	11.15	54.65

## References

[1] Kaur H., Ahuja M., Kumar S., Dilbaghi N. (2012). Carboxymethyl tamarind kernel polysaccharide nanoparticles for ophthalmic drug delivery. Int. J. Biol. Macromol..

[2] Andriamanantena R.W., Artaud J., Gaydou E.M., Iatrides M.C. (1983). Fatty acid and sterol compositions of Malagasy Tamarind Kernel Oils. JAOCS.

[3] Kaur H., Yadav S., Ahuja M., Dilbaghi N. (2012). Synthesis, characterization and evalution of thiolated tamarind seed polysaccharide as a mucoadhesive polymer. Carbohydr. Polym..

[4] Saettone M.F., Burgalassis S., Giannaccini B., Boldrini E. (2000). Ophthalmic Solutions Viscosified with Tamarind Seed Polysaccharides.

[5] Jana S., Saha A., Nayak A.K., Sen K.K., Basu S.K. (2013). Aceclofenac-loaded chitosan-tamarind seed polysaccharide interpenetrating polymeric network microparticles. Colloids Surf. B Biointerfaces.

[6] Goyal P., Kumar V., Sharma P. (2007). Carboxymethylation of Tamarind kernel powder. Carbohydr. Polym..

[7] Singh R., Malviya R., Sharma P.K. (2011). Extraction and Characterization of Tamarind Seed Polysaccharide as a Pharmaceutical Excipient. Pharmacogn. J..

[8] Sahoo S., Sahoo R., Nayak P.L. (2010). Tamarind Seed Polysachharide: A Versatile Biopolymer for Mucoadhesive Applications. JPBMS.

[9] Berretta G.U., Balzano F., Vanni L., Sansò M. (2013). Mucoadhesive properties of tamarind-seed polysaccharide/hyaluronic acid mixture: A nuclear magnetic resonance spectroscopy investigation. Carbohydr. Polym..

[10] Singh P.P. (1973). The oxalic acid content of Indian foods. Qual. Plant. Mater..

[11] Kaewkumsan P., Honggr J., Sawadee B. (2014). The use of tamarind kernel powder substitute commercial pectin. Khon Kaen Agric. J..

[12] Chandramouli Y., Firoz S., Vikram A., Mahitha B., Yasmeen B.R., Hemanthpavankumar K. (2012). Tamarind seed polysaccharides (TSP)-An adaptable excipient for novel drug delivery system. IJPPDR.

[13] Khanna M. (1997). Polyose from seeds of *Tamarindus indica* of unique property and immense pharmaceutical use. Trends Carbohydr. Chem..

[14] Sattle A., Agrawal S. (2012). Solubility enhancement potential of Tamarind seed polysaccharide as pharmaceutical excipient. Int. J. Pharm. Biol. Arch..

[15] Singh D., Wangchu L., Moond S.K. (2007). Processed products of Tamarind. Nat. Prod. Radiance.

[16] Sudjaroen Y., Haubner R., Wurtele G., Hull W.E. (2005). Isolation and structure elucidation of phenolic antioxidants from Tamarind (*Tamarindus indica* L.) seeds and pericarp. Food Chem. Toxicol..

